# A critical assessment of interatomic potentials for modelling lattice defects in forsterite Mg$_2$SiO$_4$ from 0 to 12 GPa

**DOI:** 10.1007/s00269-021-01170-6

**Published:** 2021-11-11

**Authors:** Pierre Hirel, Jean Furstoss, Philippe Carrez

**Affiliations:** Univ. Lille, CNRS, INRAE, Centrale Lille, UMR 8207 - UMET - Unité Matériaux et Transformations, F-59000 Lille, France

**Keywords:** Numerical simulation, Forsterite, Lattice defects

## Abstract

Five different interatomic potentials designed for modelling forsterite Mg$_2$SiO$_4$ are compared to *ab initio* and experimental data. The set of tested properties include lattice constants, material density, elastic wave velocity, elastic stiffness tensor, free surface energies, generalized stacking faults, neutral Frenkel and Schottky defects, in the pressure range $0-12$ GPa relevant to the Earth’s upper mantle. We conclude that all interatomic potentials are reliable and applicable to the study of point defects. Stacking faults are correctly described by the THB1 potential, and qualitatively by the Pedone2006 potential. Other rigid-ion potentials give a poor account of stacking fault energies, and should not be used to model planar defects or dislocations. These results constitute a database on the transferability of rigid-ion potentials, and provide strong physical ground for simulating diffusion, dislocations, or grain boundaries.

**Supplementary Information:**

The online version contains supplementary material available at 10.1007/s00269-021-01170-6.

## Introduction

Olivine (Mg,Fe)$_2$SiO$_4$ is the most abundant mineral in Earth’s upper mantle, as it constitutes more than 60% of its mass. As such, knowledge of its properties and response to mechanical solicitation is key to understanding the rheology of the Earth interior. Although the bulk properties of crystalline olivine are well constrained, the elementary mechanisms associated with its deformation remain challenging to characterize, and the respective roles of various defects, dislocations, grain boundaries, vacancies and impurities, are still the matter of ongoing debates (Demouchy 2021).

In complementarity with experimental work, numerical simulations at the atomic scale are a powerful tool to investigate the individual role of defects. However, modelling defects often requires large-scale models counting from a thousand to several millions of atoms, a scale that is out of reach of *ab initio* methods, and will remain so in the foreseeable future. Faster methods have to be considered, such as classical molecular dynamics simulations using empirical potentials. Such methods are reputed less accurate than *ab initio* methods, and of variable accuracy depending on the properties they were fitted to. This is why it is critically important to assess the domain of validity of an empirical potential, and make sure that it describes accurately the key properties of the material, before using it in large-scale simulations.

Over the years, various empirical interatomic potentials were developed for modelling silicate minerals and glasses. In this work we compare five different interatomic potentials parametrized over the last 40 years either specifically for forsterite, or for various silicate phases. Using experimental and *ab initio* data from literature as reference, we test the ability of the interatomic potentials to reproduce correctly some target properties of forsterite, such as the lattice parameters, elastic constants, stacking fault energies, and Schottky defects energies. The goal of this work is to provide physical ground justifying the usage of such empirical potentials for modelling defects and deformation of forsterite, in particular point defects and diffusion, dislocation glide, or grain boundaries.

## Methods and models

### Forsterite crystallography

**Fig. 1:**
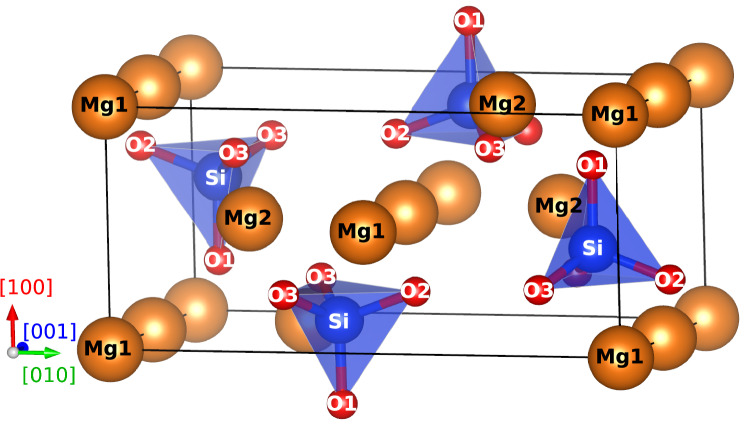
Unit cell of forsterite counts four formula units of Mg$_2$SiO$_4$, i.e., 28 atoms. Mg ions are displayed as large orange spheres, silicon as medium blue, and oxygen as small red spheres. Si$-$O bonds and SiO$_4$ tetrahedra are also represented. Atoms are labelled according to the crystallographic site they occupy

Forsterite Mg$_2$SiO$_4$ is an orthorhombic crystal, thermodynamically stable from ambient pressure up to about 12 GPa (Ringwood 1975; Hazen 1976). In the following, we describe it in the *Pbnm* space group, where the three shortest lattice vectors are such that $[100]< [001] < [010]$. The unit cell counts four formula units, *i.e.*, 28 atoms, and is represented in Fig. 1. As noticed by Bragg and Brown in their determination of the olivine structure in 1926 (Bragg et al. 1926), the oxygen ions form an approximately hexagonal close packed (hcp) sublattice. Silicon ions sit at the center of oxygen tetrahedra, and magnesium ions occupy two different sites labelled Mg1 and Mg2. Oxygen ions occupy three different types of sites labelled O1, O2, O3, such that the Si$-$O1 bonds are aligned with [100], the O3$-$O3 bonds with [001], and the O2 ion occupies the last corners of the tetrahedra. As the starting structure, we use the lattice parameters and ions positions determined by Baur in 1972 using X-ray diffraction (Baur 1972), which are available at the crystallography open database as a crystallographic information file (CIF entry 9000267). This initial structure is then relaxed using the empirical potentials as explained below.

### Semi-empirical potentials

We consider semi-empirical potentials published in literature and relying on physically sensible functions. All potentials rely on the Coulomb interaction, and differ in the charges attributed to the ions (formal or partial charges), and in the functions used to describe short-range interactions (Buckingham, Morse, 3-body...). In order to model charge-neutral defect clusters, we consider only potentials, where the charge of ions is an integer multiple of the charge of an oxygen ion, i.e., $q_\text {Mg} = q_\text {Si}/2 = -q_\text {O}$.

In 1987 Price, Parker and Leslie proposed a three-body potential for forsterite named THB1 (Price et al. 1987). It relies on formal ion charges ($q_\text {O} = -2e$), and describes short-range interactions with a Morse function and an additional three-body term to account for the covalency of Si$-$O bonds. Oxygen ions are treated in the framework of a polarizable shell model, where the core and shell of each ion do not interact through the Coulomb force, but only through a parametrized harmonic spring force. The potential parameters were obtained from Hartree–Fock calculations and experimental elastic data on rock-salt magnesium oxide MgO and quartz SiO$_2$ (Price et al. 1987). It is noteworthy that the fitting procedure included no data about forsterite itself. The THB1 potential has a long history of successful applications, including the equation of state of forsterite (Choudhury et al. 1989), high-pressure phase transitions (Guyot and Reynard 1992), vacancy formation (Jaoul et al. 1995), and the modelling of dislocations (Mahendran et al. 2017).

For all its successes, the THB1 potential is not without drawbacks. Few modern simulation codes actually implement it, such as GULP (Gale 1997) or DL_POLY (Todorov et al. 2006). Mahendran and co-workers implemented this potential into LAMMPS (Mahendran et al. 2017), with the limitation that the Coulomb interaction is computed with the Wolf summation method. Up to this date, this implementation was not merged into the main version of LAMMPS, which limits its usage. Moreover, the THB1 potential is computationally demanding, owing to the three-body term and to the fact that each oxygen ion is described as two interacting particles; as a result, modelling the unit cell with THB1 requires defining 44 particles. Finally, the THB1 potential is by design only suited to model Mg–Si–O systems at the exclusion of any other atomic species, and is very difficult to fit to other elements. Some groups modified the THB1 potential using a breathing shell model (Blanchard et al. 2005), or using different charges for oxygen ions depending on the site they occupy (Urusov and Dudnikova 2011), however such models have essentially the same limitations as the original THB1 potential. All these difficulties combined are reason enough for seeking other types of potentials, which are implemented and readily available in up-to-date simulation codes, computationally more efficient, and include the interaction parameters for various atomic species.

**Fig. 2:**
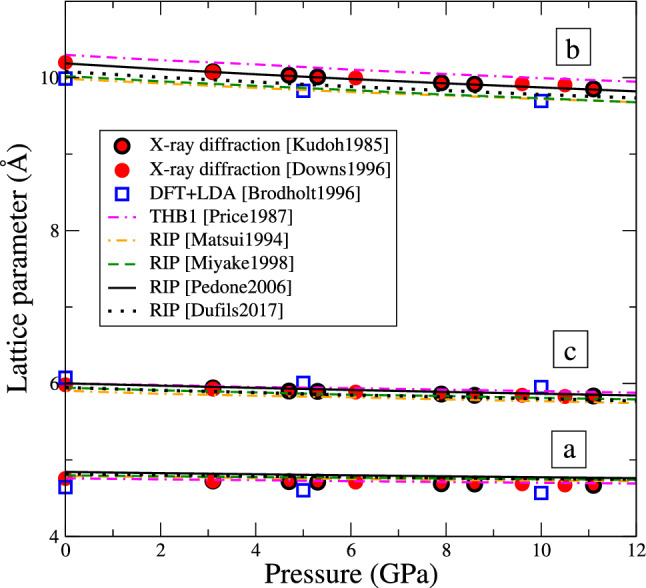
Variation of the three lattice constants of forsterite Mg$_2$SiO$_4$ as function of pressure. Values from experiments (Takeuchi and Y. 1985; Downs et al. 1996) (filled discs) and DFT calculations (Brodholt 1997) (empty squares) from the literature serve as reference. The lines are the values computed with the different interatomic potentials: the shell-model potential THB1 (pink point-double dashed), and rigid-ion potentials (RIP) Matsui1994 (orange point-dashed), Miyake1998 (green dashed), Pedone2006 (continuous black), and Dufils2017 (black points)

In 1994, Matsui designs a new rigid-ion potential (RIP) describing crystals and melts in the system CaO–MgO–Al$_2$O$_3$–SiO$_2$ (Matsui 1994). Contrary to shell-model potentials (such as THB1), rigid-ion potentials treat all ions as point charges and do not account for ion polarization. The Matsui1994 potential uses partial charges ($q_\text {O} = -0.945e$), and a Born function to describe short-range interactions. The parameters were fitted to experimental lattice parameters and bulk moduli of 26 different crystals, including MgO with the rock-salt structure, alumina Al$_2$O$_3$, different polymorphs of quartz SiO$_2$, forsterite, spinel and more. In this data set, various crystal types are represented including high-pressure phases, with various coordination including SiO$_4$ tetrahdra and SiO$_6$ octahedra. The Matsui1994 potential was applied to model liquids (Spera et al. 2011), amorphous phases (Shimoda and Okuno 2006; Tane et al. 2011), and recrystallisation in complex systems (Rymzhanov et al. 2019).

In 1998, Miyake and co-workers, unsatisfied by the inadequacy of previous potentials for describing feldspars and pyroxenes, propose another interatomic potential for modelling crystals in the K$_2$O–Na$_2$O–CaO–MgO–Al$_2$O$_3$–SiO$_2$ system (MIYAKE 1998). They also use partial charges ($q_\text {O} = -0.96e$), and a combination of a Born and a Morse functions to describe short-range interactions. The function parameters were fitted so as to reproduce the lattice constants and thermal expansion coefficients of various crystals, including forsterite.

Yet another RIP is proposed by Pedone and co-workers in 2006 (Pedone et al. 2006). It uses partial ion charges ($q_\text {O} = -1.2e$), a short-range Morse function, and a repulsive $r^{-12}$ term akin to a truncated Lennard–Jones function. In addition to Mg, Si and O, the potential includes interactions for iron in two oxidation states, along with a number of other elements. Parameters were fitted to experimental data that included lattice parameters, elastic constants, high-frequency and static dielectric constants, lattice energy, piezoelectric constants, and phonon frequencies of binary oxides. The authors then validated their parametrization by computing the bulk properties (lattice parameters and elastic constants) of ternary and quaternary crystals, including forsterite, fayalite Fe$_2$SiO$_4$, garnets, and more. So far, the Pedone2006 potential was cited in over 300 published articles, and found successful applications in a large variety of topics, such as silicate glasses (Urata 2019), recrystallization (Hu et al. 2010), grain boundaries in strontium titanate (Ramadan and De Souza 2016), thermal conductivity (Severin and Jund 2017), lithium-ion and sodium-ion battery materials (Deng et al. 2018; Lee et al. 2021), and more.

Finally, most recently Dufils and co-workers developed a new RIP, using the same partial charges as the Matsui1994 potential ($q_\text {O} = -0.945e$), and a mixture of a Buckingham function and a Gaussian function for short-range interactions (Dufils et al. 2017). This potential was specifically designed and fitted to describe melts, and not at all intended for modelling crystalline structures, nonetheless we include it in our comparison to assess its transferability to crystalline forsterite.

### Atomistic simulations

We perform simulations with the general utility lattice program (GULP) (Gale 1997), where Coulomb interactions are computed using the Ewald sum method; and with the large-scale atomic/molecular massively parallel simulator (LAMMPS) (Plimpton 1995), where Coulomb interactions are computed with the particle–particle particle–mesh (pppm) method (Eastwood et al. 1980). For calculations based on the THB1 potential, we use the custom version of LAMMPS modified by Mahendran (Mahendran et al. 2017), where the Coulomb interaction is computed with the Wolf summation method (Wolf 1995). Preliminary tests allowed us to verify that the different codes and methods yield the same lattice constants and energies.

Pressure is imposed by rescaling the simulation box and monitoring the stress tensor as computed by LAMMPS. The cell angles are constrained to 90$^\circ$, and the geometry and atom positions are optimized several times until the target pressure is reached and all forces are smaller than 10$^{-9}$ eV.Å$^{-1}$. Supercells containing lattice defects are constructed with Atomsk (Hirel 2015), and atomic structures are visualized with VESTA (Momma and Izumi 2011).

## Bulk forsterite properties

Interatomic potential functions are fitted to reproduce bulk properties, hence they are expected to match closely the DFT and experimental data. For each potential, we performed a full relaxation of atom positions and cell dimensions.

### Lattice constants and density

Forsterite is orthorhombic and therefore has three independent lattice constants, such as $a< c < b$ in the *Pbnm* space group. Figure 2 shows the resulting lattice constants as function of pressure. Experimental data from literature appears as red circles, while blue squares are results from density functional theory (DFT) calculations. The latter were performed using the local density approximation (LDA), which is renown for systematically underestimating lattice constants. The present results obtained with interatomic potentials are represented as continuous curves. All potentials reproduce the lattice constants with a very good fidelity at all pressures in the range from 0 to 12 GPa, with deviations smaller than 5% from experiment. The Pedone2006 potential matches experimental values the closest.

**Fig. 3:**
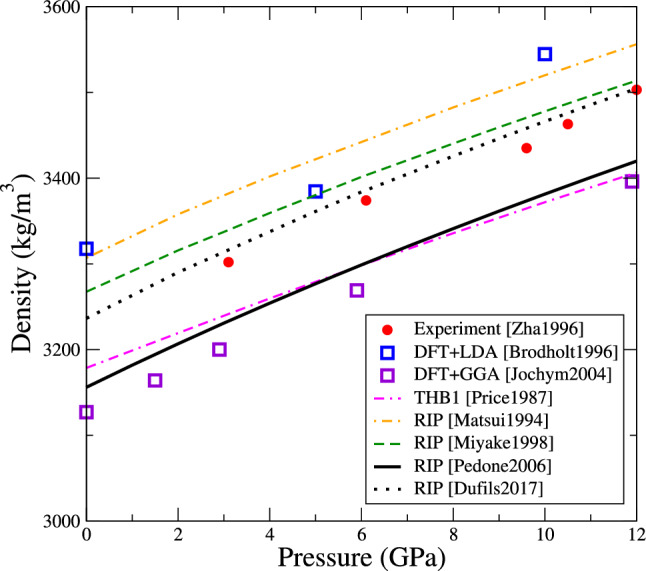
Variation of the density of forsterite Mg$_2$SiO$_4$ as function of pressure (same colour code as Fig. 2)

**Fig. 4:**
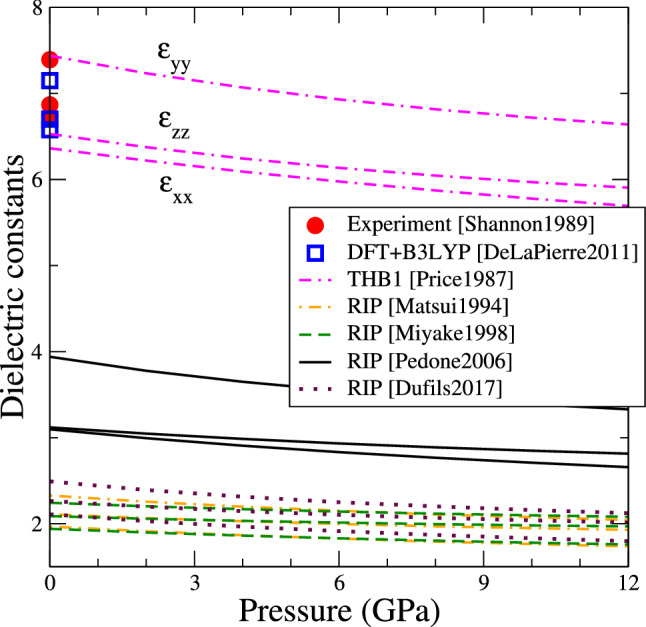
Variation with pressure of the static dielectric constants of forsterite obtained with the various interatomic potentials (same colour convention as Fig. 2). At 0 GPa experimental values are taken from Ref. (Shannon and Subramanian 1989), and DFT values from Ref. (De La Pierre et al. 2011). All values follow the ordering $\epsilon _{xx}< \epsilon _{zz} < \epsilon _{yy}$

Knowing the lattice constants, the material density is easily obtained. Figure 3 shows the density produced by the various potentials as function of pressure, compared with experimental and DFT data. As the LDA underestimates lattice constants, it overestimates the material density. Inversely, the generalized-gradients approximation (GGA) overestimates lattice constants, and thus underestimates the density. All interatomic potentials lie within these two bounds. The THB1 and Pedone2006 potentials are closest to DFT+GGA, the Matsui1994 is closest to DFT+LDA, while the Miyake1998 and Dufils2017 potentials are closest to experimental values.

### Static dielectric constants

The dielectric constants control the screening of the interactions between charges (including charged defects). Since forsterite has an orthorhombic symmetry, its dielectric response is anisotropic and characterized by a dielectric tensor, where only diagonal elements $\epsilon _{xx}$, $\epsilon _{yy}$ and $\epsilon _{zz}$ are non-zero. When using an interatomic potential, the values of dielectric constants depend on the effective ion charges that are used.

We compute the static dielectric constants of forsterite using GULP implementing the interatomic potentials described above. We compare our results with reference values from experiments of Shannon and co-workers (Shannon and Subramanian 1989) and from DFT calculations employing a B3LYP hybrid exchange-correlation functional performed by De La Pierre et al. (De La Pierre et al. 2011), both in absence of pressure. To the best of our knowledge, the dielectric constants of forsterite were not measured nor calculated at high pressure.

Figure 4 presents the static dielectric constants of forsterite as function of pressure. All potentials reproduce correctly the relative ordering $\epsilon _{xx}< \epsilon _{zz} < \epsilon _{yy}$, with only a weak anisotropy between the three coefficients, in agreement with the experimental (Shannon and Subramanian 1989) and DFT (De La Pierre et al. 2011) values, although the absolute values are not correctly reproduced. Reference values from literature are in the range of 6.5$-$7.2, with a good agreement between experiment and DFT calculations.

The (low-frequency or static) dielectric response of an ionic solid comes from two contributions: ionic displacements, and electronic structure. In the THB1 potential, if an electric field is applied then the ions are displaced, and the shells mimic the response due to the electron clouds, so that this potential accounts for both contributions. We find indeed that only the THB1 potential offers an accurate description of the dielectric constants, demonstrating that the polarizability of individual ions plays an important role. On the contrary, rigid-ion potentials can only account for the ionic contribution and miss the electronic contribution entirely. As a result, they systematically underestimate the dielectric constants. We note that the smaller the partial charge in these potentials, the weaker the dielectric constant: the Pedone2006 potential ($q_\text {O} = -1.2e$) underestimates the dielectric constants by a factor of 2, while the other RIP ($q_\text {O} \approx -0.9e$) underestimate them by a factor of 3 approximately.

### Elastic constants

**Fig. 5:**
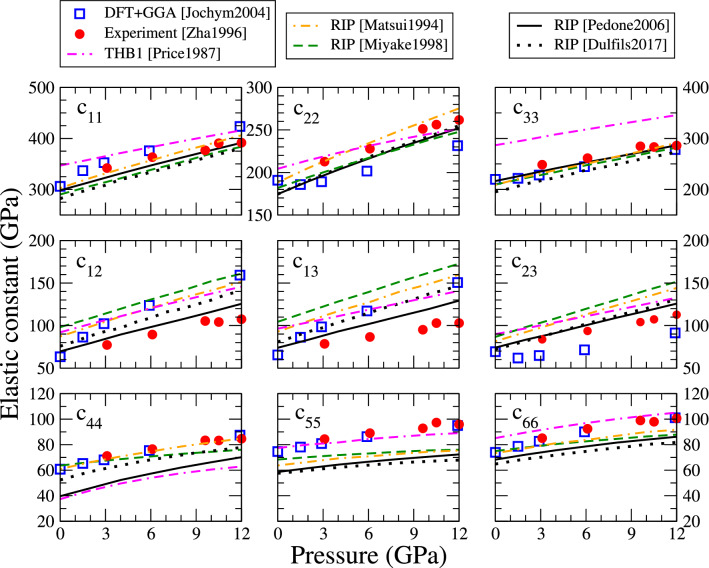
Variation with pressure of the nine independent elastic constants of forsterite computed with interatomic potentials and compared with values from literature (same colour code as Fig. 2)

The elastic properties are often a critical point when modelling the material, thus we computed the elastic tensor produced by each potential. We apply a given strain tensor $\epsilon _{kl}$ to the unit cell, and the internal stress tensor $\sigma _{ij}$ is computed by deriving forces from the empirical potential. Deformations of 2% or below are used, and we checked that the results were unchanged for deformations between 0.5% and 2%. The elastic constants are then computed according to Hooke’s law:

1 $$\begin{aligned} \sigma _{ij} = c_{ijkl} \ \epsilon _{kl} \end{aligned}$$

Forsterite has an orthorhombic symmetry, therefore it is characterized by nine independent elastic constants. Their values are reported as function of pressure in Fig. 5, again compared with experimental (red discs) and DFT (blue squares) data. Overall the interatomic potentials follow the correct trends, with deviations comparable to the differences between experimental and DFT data. Surprisingly the THB1 potential tends to systematically overestimate the $c_{33}$ component by about 30%, and to underestimate the $c_{44}$ component.

Knowing the elastic tensor allows computing the theoretical sound wave velocities in the crystal. We use the Voigt definition of the bulk and shear modulus, respectively (Hill 1952):

2 $$\begin{aligned} K= & {} \frac{ c_{11} + c_{22} + c_{33} + 2 \left( c_{12} + c_{23} + c_{13} \right) }{9} \end{aligned}$$

3 $$\begin{aligned} G= & {} \frac{ c_{11} + c_{22} + c_{33} - \left( c_{12} + c_{23} + c_{13} \right) }{15} + \frac{ c_{44} + c_{55} + c_{66} }{5} \end{aligned}$$

Assuming an homogenous medium, the velocities of longitudinal (P) and transverse (S) elastic waves are computed, respectively, as

4 $$\begin{aligned} v_P = \sqrt{ \frac{K + 4G/3}{\rho } } \ ; \ v_S = \sqrt{ \frac{G}{\rho } } \end{aligned}$$

**Fig. 6:**
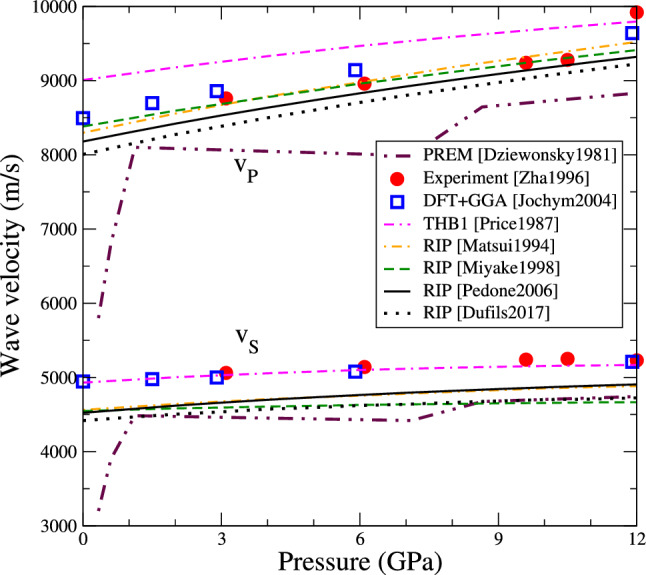
Evolution of seismic wave velocities as function of pressure (same colour code as Fig. 2). $v_P$ indicates the velocity of longitudinal (P) waves, $v_S$ those of transverse (S) waves. The colour code is the same as in Fig. 2, experimental data is taken from ref. (Zha et al. 1996), DFT data from ref. (Jochym et al. 2004), and data from the preliminary reference Earth model (PREM (Dziewonski and Anderson 1981)) are also shown for comparison

The resulting velocities are reported in Fig. 6 as function of pressure, and also compared with results from literature. The transverse velocity $v_S$ obtained with the THB1 potential matches closely experiments and DFT, while other interatomic potentials underestimate it. For the longitudinal wave velocity $v_P$, the THB1 potential overestimates its value, while other potentials are in better agreement with experimental and DFT data.

Figure 6 also shows the velocities obtained experimentally for the upper mantle in the framework of the preliminary reference Earth model or PREM (Dziewonski and Anderson 1981). Although the upper mantle is actually composed of various minerals at high temperature, we find that the velocities obtained for pure forsterite from 0 K simulations are of the same order of magnitude as those obtained in the PREM.

Overall, we find that all interatomic potentials tested reproduce with a good fidelity the bulk properties of forsterite. Depending on the target property, some potentials perform better than others, but no potential fails critically when applied to bulk, defect-free forsterite. Even the Dufils2017 potential, which was designed for modelling melts, describes very well crystalline forsterite.

### Ultimate strength

**Table 1:** Ultimate mechanical properties (in GPa) of forsterite at ambient pressure, computed with the various interatomic potentials. DFT data by Gouriet et al. serve as reference (Gouriet et al. 2019). Numbers in parenthesis give the corresponding ultimate strain

	DFT(Gouriet et al. 2019)	THB1	Matsui1994	Miyake1998	Pedone2006	Dufils2017
Ideal tensile stress (ITS) and strain						
[010]	12.1 (11.5%)	16.1 (15%)	12.8 (13%)	14.2 (13%)	10.9 (10.1%)	10.2 (9.6%)
[001]	15.9 (16%)	19.1 (13%)	16.2 (13%)	17.2 (14.2%)	16.2 (12.5%)	13.0 (10%)
[100]	29.3 (13%)	26.9 (9.6%)	23.0 (10.5%)	27.8 (13%)	20.9 (9.1%)	22.5 (10.5%)
Ideal shear stress (ISS) and strain						
(010)[001]	5.3 (18%)	7.7 (27.2%)	6.0 (21.5%)	7.1 (14.5%)	4.34 (23%)	5.1 (18.5%)
(001)[010]	6.2 (20%)	7.6 (23.5%)	6.1 (17.5%)	7.1 (15.5%)	6.34 (17.8%)	5.8 (18.8%)
(010)[100]	8.5 (18%)	17.4 (23%)	20.4 (24%)	16.1 (19.6%)	11.23 (11.8%)	15.0 (10.5%)
(100)[010]	9.0 (20%)	15.2 (23.1%)	13.2 (24.2%)	12.62 (19.6%)	11.51 (18.6%)	10.0 (16.6%)
(100)[001]	11.2 (26%)	14.0 (30%)	11.05 (25.5%)	11.4 (22.6%)	8.21 (18.1%)	8.5 (18.6%)
(001)[100]	13.4 (29.5%)	21.3 (30%)	15.7 (27.5%)	13.0 (22.6%)	10.13 (18.5%)	10.8 (19.5%)

Pursuing beyond the small deformations and elasticity, applying large strains to probe the limits of the stability of the forsterite lattice also provides a good testing bench of interatomic potentials. Instability is reached when the forsterite structure is no longer stable, which typically is associated with a maximum stress or ideal stress.

The ideal tensile and shear stresses in forsterite were recently computed by means of DFT+GGA calculations by Gouriet et al. (Gouriet et al. 2019), which we use here as a reference. A unit cell of forsterite (optimized with the relevant interatomic potential) is deformed, either in tension or in simple shear, by increments of 0.5% up to 40%. After each deformation increment, atom positions are relaxed, and the stress is derived from the forces acting on atoms.

Table 1 summarizes the ideal tensile stress (ITS) and ideal shear stress (ISS) obtained with the various potentials, and compared with DFT results from Gouriet et al. (Gouriet et al. 2019). Concerning the ITS, all potentials agree qualitatively with the DFT data on the relative ordering $[010]< [001] < [100]$. Quantitatively, the potentials maintain the forsterite structure up to tensile stresses that are higher than DFT by up to 30%. Only the Dufils2017 potential becomes unstable at lower stresses. The trend is the same concerning the ideal shear stresses (ISS): interatomic potentials support stresses that are higher than the ISS predicted by DFT.

The fact that interatomic potentials produce ideal stresses so different from DFT values is not catastrophic for their usability. The ideal stress is only a measure of how much deformation the model can sustain and still maintain a mechanically stable forsterite structure. In the end, all potentials can sustain very large strains and stresses before showing instabilities or large deviations from DFT, which is a good indicator of their robustness (see Supplementary Material).

## Free surfaces

**Table 2:** Free surface energies (J.m$^{-2}$) in forsterite Mg$_2$SiO$_4$ computed with the empirical potentials, and compared with DFT results from Bruno et al. (Bruno et al. 2014). Numbers in parenthesis give the deviation from DFT data

Surface	DFT(Bruno et al. 2014)	THB1	Matsui1994	Miyake1998	Pedone2006	Dufils2017
(010)	1.22	1.25 (+2.5%)	1.12 ($-8.2$%)	1.11 ($-9.0$%)	1.13 ($-7.4$%)	0.89 ($-27$%)
(120)	1.36	1.58 (+16.2%)	1.28 ($-5.9$%)	1.23 ($-9.6$%)	1.37 (+0.7%)	0.99 ($-27.2$%)
(001)	1.78	1.58 ($-11.2$%)	1.40 ($-21.3$%)	1.32 ($-25.8$%)	1.52 ($-14.6$%)	1.12 ($-37$%)
(101)	1.78	1.89 (+6.2%)	1.47 ($-17.4$%)	1.42 ($-20.2$%)	1.58 ($-11.2$%)	1.18 ($-33.7$%)
(111)	1.84	1.79 ($-2.7$%)	1.55 ($-15.8$%)	1.50 ($-18.5$%)	1.67 ($-9.2$%)	1.21 ($-26.1$%)
(021)	1.90	1.94 (+2.1%)	1.51 ($-20.5$%)	1.48 ($-22.1$%)	1.68 ($-11.6$%)	1.24 ($-34.7$%)
(110)	2.18	2.26 (+3.7%)	1.72 ($-21.1$%)	1.73 ($-20.6$%)	1.81 ($-17$%)	1.46 ($-33.0$%)

The main motivation for using interatomic potentials is going beyond the simple bulk properties and simulating large-scale systems. In particular, modelling plastic deformation requires a good description of point defects (vacancies, interstitials, impurities), linear defects (dislocations), and planar defects (surfaces, stacking faults, grain boundaries). Unfortunately, the structure and energetics of such defects in forsterite is extremely complex and the published data scarce, which complicates the evaluation of the suitability of interatomic potentials to describe such defects.

As a first defect, we consider the free surfaces of forsterite. Their energy controls the shape and morphology of olivine crystals, and are also strongly related to interface and grain boundary energies, hence it is of critical importance to assess the ability of interatomic potentials to describe them correctly. Recently, Bruno et al. performed DFT calculations of surface energies in forsterite (Bruno et al. 2014). We use their published configurations, relaxed with DFT, as the initial configurations for our own simulations. Each configuration is relaxed (ions and box geometry in the plane of the free surface) using the empirical potentials, and the surface energy is computed:

5 $$\begin{aligned} \gamma _{\text {surf}} = \frac{E^{\text {surf}} - E^0}{2 S} \end{aligned}$$

where $E^{\text {surf}}$ is the total energy of the cell containing free surfaces, $E^0$ the total energy of an equivalent bulk and 3-D periodic cell of forsterite with the same number of atoms, and *S* the area of the free surface. The factor of 2 accounts for the fact that the cell contains two equivalent free surfaces. These calculations are performed only at 0 GPa, because free surfaces are not expected to form in the mantle at high pressure, and the constraints to model high-pressure free surfaces are not defined unambiguously.

Table 2 gives the free surface energies computed with the interatomic potentials, along with the DFT results from Bruno et al. (Bruno et al. 2014). All potentials give correctly the (010) surface as the most favourable, and the (110) surface as the least favourable. Differences appear in the relative energies of the other surfaces. Overall, the THB1 potential is the best match in absolute values, but gives a wrong ordering $(111)<(101)$, while the other potentials agree with DFT on the ordering $(101)<(111)$. The other potentials underestimate the values of the surface energies by 10 to 20%, and the Matsui1994 and Miyake1998 potentials both invert the ordering of the (021) and (111) surfaces. The Pedone2006 and Dufils2017 potentials both give the correct energy ordering for all tested surfaces, but the latter largely underestimates the energies by about 30%.

Overall, the Pedone2006 potential is the best match to DFT data when considering both the absolute and relative values of the surface energies.

## Generalized stacking faults

A second important class of defects are the stacking faults. The most complete and reliable data on this topic come from Durinck et al., who used DFT with GGA to compute the energy density of generalized stacking faults (GSF) in various slip planes of forsterite, at 0 and 10 GPa (Durinck et al. 2005). This allows killing two birds with one stone: first, the GSF are closely related to the atomic core structure of dislocations (Vitek 1968), and can be used to predict the lattice resistance to dislocation glide (Denoual 2004). Second, sampling the GSF means evaluating the energy of a great number of unstable configurations. We argue that if an interatomic potentials reproduces accurately the GSF, then it would also be well suited to model other types of planar defects, including grain boundaries.

We used the different interatomic potentials to compute the GSF in forsterite, at imposed pressures of 0 and 10 GPa to compare with the previously mentioned DFT results from literature. The unit cell is duplicated 16 times in the direction normal to the plane of interest, and the stacking fault is constructed by translating the top part of the crystal by a vector $\tau$ contained in the given plane, all while maintaining the integrity of SiO$_4$ tetrahedra (i.e., without shearing or cutting Si–O bonds), similar to the work by Mahendran et al. (Mahendran et al. 2017). Mg and Si ions are constrained to relax only in the direction normal to the fault, while oxygen ions (and their shells in the case of THB1) are free to relax in all directions.

**Fig. 7:**
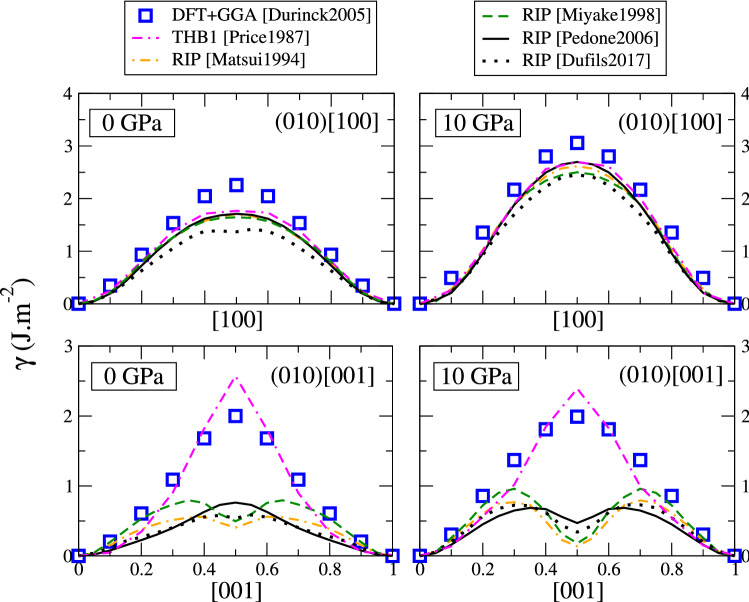
Generalized stacking fault energy density in forsterite, in the (010) plane, along the [100] direction (top) and [001] direction (bottom), at 0 GPa (left) and 10 GPa (right). DFT data (blue squares) from Ref. (Durinck et al. 2005)

We begin with the stacking faults in the (010) plane, reported in Fig. 7. Along the [100] direction, DFT (blue squares) produces a single maximum, meaning that dislocations belonging to the (010)[100] slip system have a compact core structure. All potentials follow the same qualitative behaviour, although they all tend to underestimate the fault energies. Overall the agreement can be considered satisfactory, and potentials can be expected to produce correct or reasonable dislocation core structures.

The situation is radically different along the [001] direction. There again, DFT predicts a single maximum for a shift vector 1/2[001], which leads to compact dislocation cores (Durinck et al. 2005). Unfortunately, most interatomic potentials fail to reproduce this behaviour. The most accurate is the THB1 potential, which is in very good agreement with DFT at both pressures investigated. This was already verified by Mahendran et al., and justifies the use of this potential to model dislocations in forsterite (Mahendran et al. 2017). The Pedone2006 potential (continuous black curve) largely underestimates the SF energies, meaning that the dislocations Peierls stresses may be underestimated. However at 0 GPa it produces a single maximum and thus would produce a compact dislocation core. Surprisingly, the Dufils2017 potential matches closely the behaviour of the Pedone2006 potential, and produces a single maximum. Although not perfect, these two potentials may still be useful for modelling dislocations, at the condition of taking appropriate care. By contrast, the Matsui1994 and Miyake1998 rigid-ion potentials predict a local minimum at 1/2[001], *i.e.*, a metastable stacking fault (SF). This is catastrophic, as it would result in the dissociation of (010)[001] dislocations, in disagreement with DFT and Peierls–Nabarro calculations by Durinck et al. (Durinck et al. 2007). These potentials should not be used at all to model dislocations belonging to the (010)[001] slip system. At high pressure (10 GPa), all RIP potentials including the Pedone2006 produce a metastable SF, in complete disagreement with DFT. Only the THB1 potential remains in good agreement with DFT at high pressure.

The energy maximum along [001] corresponds to a configuration, where pairs of Mg ions come close to each other at the interface. Analysis of the relaxed configurations (reported in the Supplementary Material) show that all potentials produce a similar configuration, therefore the discrepancies in energy do not come from a relaxation problem, but from the potential functions themselves. By construction, all interatomic potentials assume a Coulomb repulsion between Mg ions, and neglect short-term interaction between them. This is a reasonable approximation in bulk forsterite, where Mg ions are not first neighbours, however it is likely the source of errors when Mg ions get closer. The repulsion is then entirely controlled by the Coulomb term, i.e., by the charge carried by Mg ions. We notice that the smaller the charge in a potential, the smaller the energy of the (010)[001] stacking fault. The THB1 potential with formal charge $q_\text {Mg} = +2$ gives the largest SF energy; the Pedone2006 with partial charge +1.2 underestimates the SF energy by a factor of 2; and last, the Matsui1994 and Miyake1998 potentials (which have the smallest partial charges) produce a local energy minimum. This trend seems to indicate that the error lies in the short-range Mg–Mg interaction, which may be corrected by fitting suitable short-term parameters in the Buckingham or Morse functions of the potentials. We leave such parametrization to another work.

**Fig. 8:**
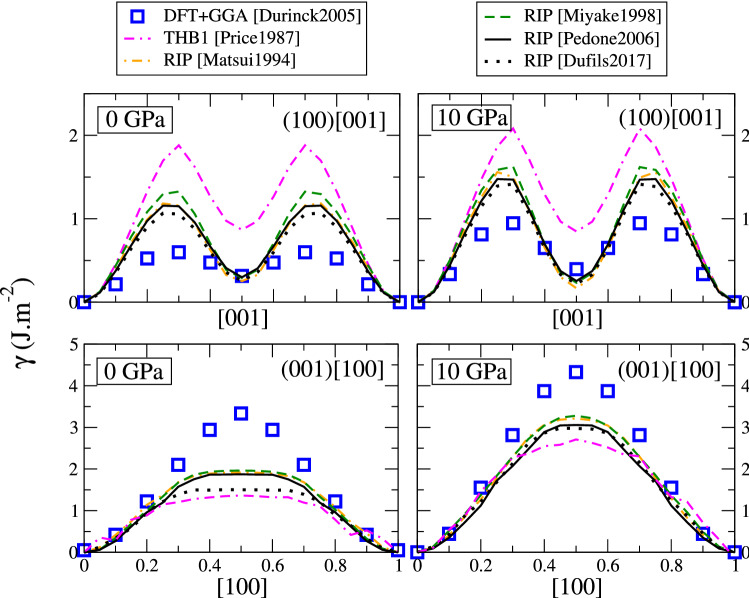
GSF energy density in forsterite, in the (100) plane along the [001] direction (top), and in the (001) plane along [100] (bottom), at 0 GPa (left) and 10 GPa (right). DFT data (blue squares) from Ref. (Durinck et al. 2005)

We also computed the GSF in the other low-index planes (100) and (001). Since dislocations with [010] Burgers vector are extremely unfavourable in forsterite, we restricted the computation to the directions relevant to the known slip systems, (100)[001] and (001)[100]. The corresponding GSF energy densities are reported in Fig. 8. All potentials are qualitatively in good agreement with DFT, in both slip planes and at both pressures considered. In the (100) plane we find a metastable stacking fault at 1/2[001] in agreement with DFT. The presence of this metastable SF means that (100)[001] dislocations modelled with those potentials would dissociate, which is in agreement with Peierls–Nabarro calculations by Durinck et al. (Durinck et al. 2007). The THB1 potential overestimates all energies by a factor of 2, which is expected to result in the underestimation of dissociation distances. Rigid-ion potentials overestimate unstable energies, but are in excellent quantitative agreement with DFT concerning the metastable stacking fault at 1/2[001].

In the (001) plane, along [100] the GSF goes through a single maximum according to both DFT and the empirical potentials. At 0 GPa, the latter produce a plateau instead of a bell-shaped curve, and the energies are underestimated. The agreement with DFT is better at 10 GPa. These discrepancies can be considered minor, all potentials can be considered in reasonable agreement with DFT data in (100) and (001) planes, and good candidates to model dislocations belonging to these slip systems.

To summarize, only the THB1 potential gives a good account of all GSF energies at all pressures investigated, even though it largely overestimates energies in the (100) plane. The Pedone2006 potential can be considered as satisfactory at ambient pressure, however it seems to fail at describing stacking faults in the (010) plane at high pressure. Other potentials produce a wrong energy landscape in the (010) plane at all pressures.

## Frenkel pairs

A Frenkel defect forms when an atom is moved from its lattice site into an interstitial site. If a Frenkel pair is introduced in a simulation cell, its energy depends on the separation distance between the vacancy and interstitial because of their respective elastic fields and the long-range Coulomb interaction. In addition, the pair forms an electric dipole, which tends to polarize the whole structure. Finally, using periodic boundary conditions, the Frenkel pair interacts with an infinite array of replica, thus complicating the evaluation of its energy.

**Fig. 9:**
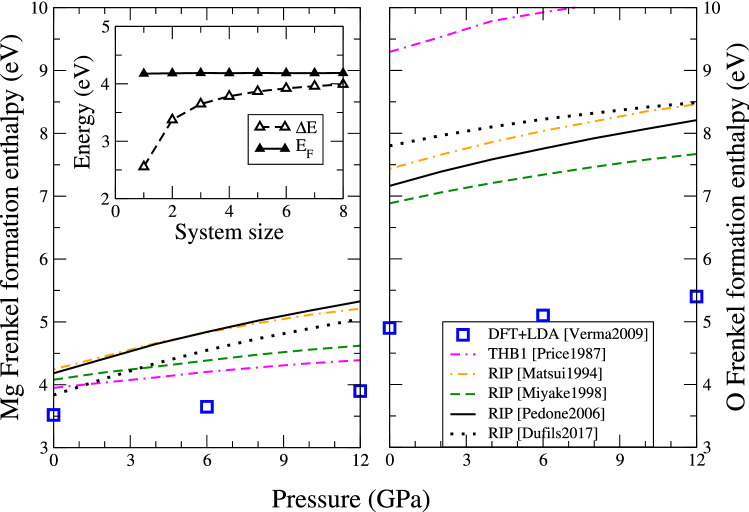
Enthalpy of formation of Mg$^{2+}$ (left) and O$^{2-}$ (right) Frenkel defects as function of pressure in forsterite. DFT data is from Ref. (Verma and Karki 2009). The inset shows the energy difference before ($\Delta$E, empty triangles) and after accounting for the correction term $E_{\text {corr}}$ ($E_F$, solid triangles) as function of the system size, obtained at 0 GPa with the Pedone2006 potential

To circumvent this, we compute separately the total energy $E_\text {v}^{N-1}$ of a system with a vacancy, and the energy $E_\text {i}^{N+1}$ of a system containing an interstitial. This is equivalent to considering that the two defects are infinitely separated. The formation energy (enthalpy) of the Frenkel pair is then computed:

6 $$\begin{aligned} H_F = E_\text {i}^{N+1} + E_\text {v}^{N-1} - 2 E_0^N - E_\text {corr} \end{aligned}$$

where $E_0^N$ is the total energy of a defect-free bulk system with *N* atoms. $E_\text {corr}$ is a correction term due to the interaction of a charged defect with its periodic replica. This contribution can be computed analytically as $E_\text {corr} = -\frac{1}{2} \alpha q^2 / (\epsilon L)$, where *L* is the typical cell size (Leslie and Gillan 1985). However the Madelung’s constant $\alpha$ depends on the cell geometry and therefore on pressure, and is difficult to obtain for an orthorhombic cell of forsterite. Instead, we use a numerical method inspired by the Ewald summation, and already used by Brodholt (Brodholt 1997). In an empty simulation cell with the same dimensions as the defective cell, we place a single ion. Using periodic boundary conditions, we compute the Coulomb interaction energy. Because the cell is empty, this situation mimics an infinite periodic array of point charges separated by vacuum, therefore we correct the Coulomb interaction by introducing the pressure-dependent dielectric constant computed with the potential and presented before. Since the dielectric constant is only weakly anisotropic, at each pressure we use the average value of all three components. The inset in Fig. 9 gives the energy as function of system size before (empty triangles) and after application of the correction $E_\text {corr}$ (filled triangles), for the Mg Frenkel pair computed with the Pedone2006 potential. Application of the correction allows for rapid convergence of the Frenkel energy even in small systems. We note that the uncorrected values appear to converge towards the corrected value for large system sizes, thus giving confidence in our computation of the correction term. The results presented below were obtained in supercells containing 896 atoms, corresponding to the system size 2 in the inset graph of Fig. 9.

Figure 9 reports the enthalpy of formation of Mg and O Frenkel pairs as function of pressure. We compare our results with those obtained by Verma and Karki using DFT calculations (Verma and Karki 2009). According to DFT, the energy of the Mg Frenkel defect rises from 3.52 eV at ambient pressure, to about 3.9 eV at 12 GPa (Verma and Karki 2009). The empirical potentials tend to overestimate these energies by about 0.5 eV, the error rising up to 1 eV at high pressure.

The error is greater on the oxygen Frenkel defect, as shown on the right-hand side of Fig. 9. Instead of formation enthalpies ranging from 5 to 5.5 eV according to DFT calculations (Verma and Karki 2009), empirical potentials largely overestimate enthalpies ranging from 7 to 8.5 eV. This may come from the fact that when an oxygen ion is missing, potentials tend to connect the defective tetrahedron with a neighbouring one, and thus two tetrahedra share an oxygen ion and are connected by their tips.

## Schottky defects

Schottky defects are neutral vacancy clusters. In forsterite, four types of Schottky defects can form: one formed of one magnesium and one oxygen vacancies (MgO partial Schottky defect); one formed of SiO$_2$ vacancies; one formed of MgSiO$_3$ vacancies; and finally, the full Schottky defect Mg$_2$SiO$_4$.

**Fig. 10:**
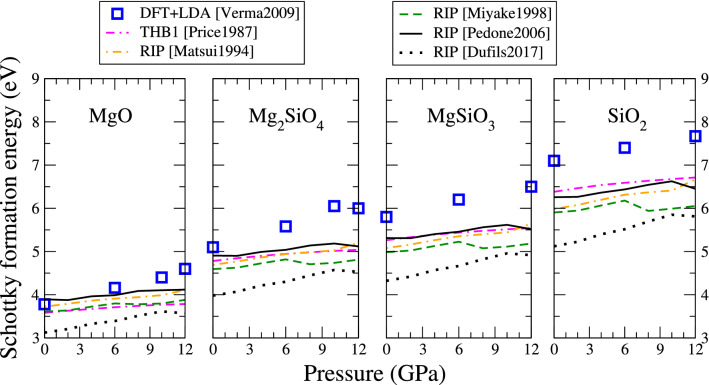
Enthalpy of formation of partial and full Schottky defects in forsterite as function of pressure. DFT data is from Ref. (Verma and Karki 2009)

As for the Frenkel defects, the DFT calculations by Verma and Karki (Verma and Karki 2009) are used as a reference. As for Frenkel defects, we compute the total energies of supercells containing a single Mg, Si or O vacancy, respectively, $E_{V_\text {Mg}}^{N-1}$, $E_{V_\text {Si}}^{N-1}$ and $E_{V_\text {O}}^{N-1}$. These energies are evaluated at different pressures from 0 to 12 GPa. The formation enthalpies of the (unbound) Schottky defects are then computed using

7 $$\begin{aligned} H_S^{\text {MgO}} = E_{V_\text {Mg}}^{N-1} + E_{V_\text {O}}^{N-1} - 2 E_0^N + \mu _\text {MgO} - E_\text {corr} \end{aligned}$$

8 $$\begin{aligned} H_S^{\text {SiO}_2} = E_{V_\text {Si}}^{N-1} + 2E_{V_\text {O}}^{N-1} - 3 E_0^N + \mu _{\text {SiO}_2} - E_\text {corr} \end{aligned}$$

9 $$\begin{aligned}&\begin{aligned} H_S^{\text {MgSiO}_3} =\;\;&E_{V_\text {Mg}}^{N-1} + E_{V_\text {Si}}^{N-1} + 3E_{V_\text {O}}^{N-1} - 5 E_0^N \\&+ \mu _\text {MgO} + \mu _{\text {SiO}_2} - E_\text {corr} \end{aligned} \end{aligned}$$

10 $$\begin{aligned}{H}_{S}^{\text{Mg}_{2}\text{SiO}_{4}} = 2E_{V_\text{Mg}}^{N-1} + E_{V_\text {Si}}^{N-1} + 4E_{V_\text {O}}^{N-1} - 7 E_0^N \\ + 2\mu _\text {MgO} + \mu _{\text {SiO}_2} - E_\text {corr} \end{aligned}$$

The correction terms $E_\text {corr}$ account for interaction of vacancies with their periodic replica and was discussed in the previous section. The terms $\mu _\text {MgO}$ and $\mu _{\text {SiO}_2}$ refer to the chemical potential of the neutral units removed from the lattice. Here we use the lattice energy of rock-salt MgO and $\alpha$-quartz SiO$_2$, respectively, as computed with the corresponding potential. The Schottky formation enthalpies therefore depend on the ability of interatomic potentials to correctly describe the parent oxides phases (see Supplementary Material). For sake of consistency, the MgSiO$_3$ partial Schottky defect is also computed assuming incorporation into MgO and SiO$_2$ phases.

Figure 10 reports the evolution of the partial and full Schottky defects in forsterite, as calculated with the different interatomic potentials, in the pressure range from 0 to 12 GPa. Qualitatively, all interatomic potentials correctly reproduce the energy ordering of Schottky defects at all pressures:

$$\begin{aligned} H_S^{\text {MgO}}< H_S^{\text {Mg}_2\text {SiO}_4}< H_S^{\text {MgSiO}_3} < H_S^{\text {SiO}_2} \end{aligned}$$

Quantitatively, the interatomic potentials are in reasonable agreement with DFT, with typical deviations smaller than 1 eV. Only the Dufils2017 potential underestimates the energies by more than 1 eV for all Schottky defects. Other interatomic potentials are in better agreement with DFT, especially for the partial MgO and for the full Schottky defects. For all defects, errors also tend to become larger as the pressure increases. It is noteworthy that the rigid-ion potentials do not deviate from DFT significantly more than the THB1 potential does.

## Performance

Finally, we benchmark the relative performance of all five interatomic potentials. Supercells of crystalline forsterite with different sizes up to 112,000 atoms are constructed. In the case of THB1 potential, a number of atoms *N* means that the system contains 11*N*/7 particles, because oxygen ions are described as cores and shells. After initializing atom velocities for a temperature of 300 K, a molecular dynamics (MD) simulation is run for 1,000 steps in the microcanonical (NVE) ensemble, using a time step of 1 fs. No dump file is written to reduce the impact of disk access. Simulations are run with LAMMPS in parallel using 4 threads, on a desktop computer equipped with an Intel Xeon E5-1620 v2 CPU running at 3.7 GHz and 16 GB of RAM.

**Fig. 11:**
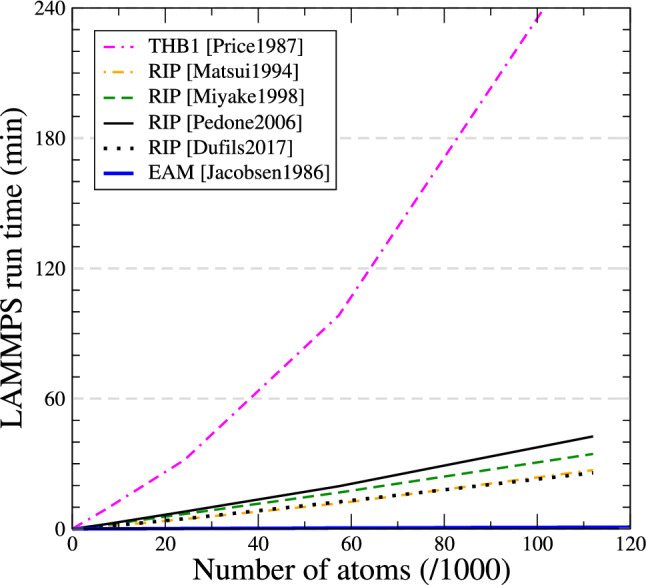
Simulation time (minutes) for running 1,000 steps of molecular dynamics with the various potentials for forsterite, as function of the number of atoms (or number of cores for the THB1 potential). Results of a similar simulation with an embedded atom potential (EAM) for aluminium are also shown for comparison (thick blue line)

Simulation times as function of number of atoms are reported in Fig. 11. The four rigid ion potentials show similar performance, requiring between 20 min (Matsui1994 and Dufils2017) and 40 min (Pedone2006) to complete the MD simulation with 112,000 atoms. The THB1 performs much more poorly, requiring no less than 4 h to complete the same simulation. Part of this performance issue may be accounted for by the shells: a system of 112,000 atoms is modelled using a total of 176,000 particles (cores+shells). However our benchmark shows that the THB1 run time increases much faster than would be anticipated just because of shells. This heavy toll comes mostly from the complexity of the potential function, which counts no less than four different pair contributions (Coulomb, Buckingham, harmonic) and a three-body term, causing the number of computed interactions to increase faster than the number of atoms.

The THB1 potential is remarkably accurate, and can be used to model moderately large systems. However, its poor performance limits its usage to a few hundred thousands of atoms at best. For million-atom systems, computational cost is largely in favour of rigid-ion potentials.

For the sake of comparison, we performed MD simulations using similar conditions on crystals of face centred cubic (fcc) aluminium, using an embedded atom method (EAM) potential to model interactions (Jacobsen et al. 1987). The run time as function of system size is also reported in Fig. 11 (thick blue line): for a system of 108,000 atoms the MD simulation runs in about 42 s, making the EAM potential at least 50 times faster than rigid ion potentials. This difference is consistent with the benchmarks published on the LAMMPS Web site (LAMMPS Web page). The relatively heavy computational cost of rigid-ion (or shell model) potentials comes mostly from the evaluation of the long-range Coulomb interaction with the pppm method. This computational cost can be reduced by decreasing the pppm accuracy, or using a different method for computing the Coulomb interaction. For instance, Wolf’s summation method can be faster than pppm, with the drawback of being sensitive to the choice of damping factor and truncation radius (Baker and Hirst 2014). No general advice can be given as ultimately, the choice of method depends on the type of atomic system, boundary conditions, defects present, and so on.

## Discussion

### Accuracy

Interatomic potentials are often fitted to bulk properties, therefore they are expected to give an excellent description of the bulk crystal. Aside from an underestimation of dielectric constants due to the use of partial charges, all interatomic potentials that we tested offer a good description of the lattice and elastic constants of pristine forsterite. This is not surprising, since their respective potential functions were fitted to experimental or *ab initio* data. Even the Dufils2017 potential, which was not designed for modelling crystalline forsterite, produces quite accurate bulk properties. It must be noted that potentials for minerals are often fitted with high-pressure properties in mind, and indeed the potentials remain robust for modelling forsterite at least up to 12 GPa, and probably at higher pressures. This is probably the reason why they are able to capture the energetics for a wide range of bond lengths.

The critical question is that of their transferability to defective systems. This is a well known limitation of interatomic potentials, and the reason why their accuracy and transferability must be tested as thoroughly as possible before applying them to complex problems. Indeed, we find that the accuracy of the potentials differ when defects are present in the system. That is expected, because defects often cause large variations in bond lengths and angles, or in the number of neighbouring atoms (coordination). Simple pair potentials are often poorly suited to capture the energetics of such drastic deviations from the perfect crystal. Nonetheless, we find that the THB1 potential successfully passed all the tests and produces defects energetics in excellent agreement with DFT calculations. The Pedone2006 potential comes close behind. Its good accuracy and performance in terms of computation time makes it an ideal candidate for modelling all types of defects, including dislocations or diffusion of point defects. Since it also gives a good description of MgO and $\alpha$-quartz SiO$_2$, it can probably be used to model interfaces between these minerals. The Pedone2006 potential appears to fail only in describing (010) stacking faults at high pressure, meaning that it would probably not give a good description of interfaces and dislocations related to this plane at high pressure.

Other potentials give good results when modelling bulk forsterite or point defects, however they fail at describing stacking faults. As a result, we advise against using them for modelling dislocations, grain boundaries, or planar defects in general in forsterite.

At last, it must be pointed out that we did not compute dynamic lattice properties, such as phonon modes. Researchers who are interested in high-temperature behaviour of forsterite should test the accuracy of the potential for dynamic properties.

### Extrinsic defects in forsterite

Although we restricted our work to native defects in forsterite, foreign elements are known to have a major impact on the properties of this phase. Iron is a major constituent of natural olivine, as it may typically count for as much as 20 wt.% of its composition (Ringwood 1975). Other elements may also be present as traces, such as Ca, Ni or Mn (see for instance the review by Demouchy (Demouchy 2021)). The intercalation of hydrogen and the formation of hydroxyl groups is also expected to occur in the mantle, and to have a significant influence on the mechanical and electrical response of olivine (Justice et al. 1982; Wang et al. 2006; Demouchy and Bolfan-Casanova 2016). However when modelling an atomic system with an interatomic potential, one is limited by the set of chemical elements that the said potential is able to describe.

The THB1 potential was initially designed for modelling forsterite Mg$_2$SiO$_4$. As such it is capable to describe interactions only between Mg, Si and O ions, at the exclusion of any other element. De Leeuw et al. extended it to include hydrogen, at the price of defining two different types of oxygen ions depending on the site they occupy (De Leeuw et al. 2000). The resulting potential has the same limitations as the THB1 as discussed above, with additional complexity and computational time. In a separate work, Blanchard and co-authors modified the THB1 potential using a breathing-shell model and adding parameters fitted for germanium, and applied it to wadsleyite (Blanchard et al. 2005). Fitting parameters for additional elements is a very heavy task, so it can be assumed that the THB1 potential will remain limited in the atomic interactions it can describe in the foreseeable future. Similarly, the Matsui1994 potential includes only calcium in addition to Mg, Si and O, which limits its reach.

The other rigid-ion potentials (RIP) that we tested were designed for describing more diverse compositions, and so include many more elements. In addition to Mg, Si and O, the Miyake1998 potential includes parameters for aluminium (Al), calcium (Ca), sodium (Na), and potassium (K). Miyake validated his potential by computing the bulk properties of 26 binary and ternary phases (MIYAKE 1998), which makes it a good candidate to investigate minerals with these compositions.

The Pedone2006 potential is by far the richest, including parameters for 28 elements. The immediate availability of parameters for iron in two oxidation states (modelled as Fe$^{1.2+}$ and Fe$^{1.8+}$) makes this potential a good candidate for investigating olivine (Mg$_{1-x}$Fe$_x$)$_2$SiO$_4$ with various iron contents, from forsterite ($x=0$) to fayalite ($x=1$). Although Pedone and co-workers validated their potential by computing the bulk properties of both phases (Pedone et al. 2006), its accuracy for intermediate compositions or defects in these phases remain untested. In addition, one must bear in mind that magnetism is unaccounted for in such empirical potentials, so magnetic effects (like those due to iron atoms) remain out of reach. The Pedone2006 potential also includes light elements such as lithium (Li$^+$), however fitting parameters for hydrogen (H$^+$) are still unavailable. Fitting parameters for hydrogen is no small task and is rendered difficult by the charge transfer in OH groups that differ from bulk forsterite, however the development of an interatomic potential that would allow modelling hydrogen binding energies and diffusion in olivine would be of great interest to the mineral physics community.

## Conclusion

We assessed the accuracy and transferability of five different empirical potentials to describe forsterite Mg$_2$SiO$_4$: the shell-model potential THB1, and four different rigid-ion potentials. The results obtained with these potentials were compared with reference data from experiments and DFT calculations from literature. We find that all potentials give a satisfactory description of bulk forsterite in its stability pressure range ($0-12$ GPa), however their accuracy is challenged when modelling defects. The THB1 potential appears to give the most accurate representation of all defects, at the cost of a greater complexity and computation time. The Pedone2006 potential comes close behind, as it gives a good description of all defects investigated in forsterite, as well as rocksalt MgO and $\alpha$-quartz SiO$_2$. Its only major failure concerns the energetics of stacking faults in the (010) plane at high pressure. The Matsui1994 and Miyake1998 potentials have similar behaviours, both failing to describe planar defects and the parent oxide phases MgO and SiO$_2$ at all pressures. The most recent Dufils2017 potential exhibits similar problems, which is not surprising as it was initially designed to describe melts and not crystalline phases. In the end, the Pedone2006 potential appears to give the best trade-off in terms of accuracy and computation time to describe defects in Mg$_2$SiO$_4$ forsterite. Our results indicate that it can be applied to a broad range of problems, such as point defects and diffusion, dislocation glide, planar defects or grain boundaries.

## Supplementary Information

Below is the link to the electronic supplementary material.

Supplementary file1 (PDF 776 KB)
