# Supplementary material for: A critical assessment of interatomic potentials for modelling lattice defects in forsterite Mg2SiO4 from 0 to 12 GPa
Source: Phys Chem Miner. 2021 Nov 11;48(12):46. doi: 10.1007/s00269-021-01170-6 (PMC8585851; doi:10.1007/s00269-021-01170-6)
Supplement: Supplementary file 1 — Supplementary file1 (PDF 776 KB) [file 269_2021_1170_MOESM1_ESM.pdf]

Supplementary Material for: A critical assessment of interatomic potentials for modelling lattice defects in forsterite Mg_2SiO_4 from 0 to 12 GPa

Pierre Hirel[a,*], Jean Furstoss[a], Philippe Carrez[a]

[a] *Univ. Lille, CNRS, INRAE, Centrale Lille, UMR 8207 - UMET - Unité Matériaux et Transformations, F-59000 Lille, France*

We present here additional material obtained from our calculations: the potential functions and fitted parameters for all five interatomic potentials compared in the main manuscript, the complete stress-strain curves obtained from deformation simulations to determine the ideal tensile and shear stresses, configurations corresponding to (010) stacking fault, and the lattice and elastic constants of the parent oxide phases MgO and SiO_2.

1. Potential parameters

Table 1 reports the potential functions, as well as the fitted parameter values, for each of the five potentials compared in our study. Compared to the initial publications, The potential functions are written so as to comply with their implementation within LAMMPS, and the parameters are in units corresponding to the "metal" units (i.e. energies in eV, distances in angströms).

2. Ideal stress curves

The computation of the ideal tensile and shear stresses required to deform the samples and monitor their internal stress. In this section we present the complete deformation curves obtained with the different interatomic potentials, and compare them with previous DFT calculations by Gouriet et al. (Gouriet et al., 2019).

Fig. 1 shows the stress-strain curves obtained after pure tensile deformation. All interatomic potentials have the same slope at the origin as the DFT curve, which correspond to the stiffness constants c_{11}, c_{22} and c_{33}. As discussed in the manuscript the THB1 potential overestimates the c_{33} constant, which is visible in the third graph. Along [100], interatomic potentials follow DFT with a good fidelity up to about 8% deformation (Pedone2006), or even up to 13% (Miyake1998), before reaching instability. Along [010] and [001] they match up to 10% deformation. Overall, interatomic potentials describe correctly the energy and

*Corresponding author: pierre.hirel@univ-lille.fr

THB1 (Price et al., 1987) $q_{\mathrm{O}}^{\mathrm{core}} = +0.848e$, $q_{\mathrm{O}}^{\mathrm{shell}} = -2.848e$

$$U(r_{ij}) = \frac{q_i q_j}{r_{ij}} + A_{ij} \exp\left(\frac{-r_{ij}}{B_{ij}}\right) + \frac{C_{ij}}{r_{ij}^6}$$

$U(r_{ijk}) = k^B \left(\theta_{ijk} - \theta_0\right)^2$ $U_{\mathrm{cs}} = \frac{1}{2} k^S r^2$

	A_{ij} (eV)	B_{ij} (Å)	C_{ij} (eV.Å^6)
Mg−O	1428.5	0.2945	0
Si−O	1283.9	0.3205	10.66
O−O	22764.3	0.1490	27.88

$k^B = 2.09$ eV.rad^{-2} $k^S = 74.92$ eV.Å^{-1}

Matsui1994 (Matsui, 1994) $q_{\mathrm{O}} = -0.945e$

$$U(r_{ij}) = \frac{q_i q_j}{r_{ij}} + A_{ij} \exp\left(\frac{\sigma - r_{ij}}{\rho_{ij}}\right) - \frac{C_{ij}}{r_{ij}^6}$$

	A_{ij} (eV)	ρ_{ij} (Å)	σ_{ij} (Å)	C_{ij} (eV.Å^{12})
Mg−O	0.0077454208	0.178000	2.71550	27.37509320
Si−O	0.0070056896	0.161000	2.54190	46.45755920
O−O	0.0120097536	0.276000	3.64300	85.38578984

Miyake1998 (MIYAKE, 1998) $q_{\mathrm{O}} = -0.96e$

$$U(r_{ij}) = \frac{q_i q_j}{r_{ij}} + A_{ij} \exp\left(\frac{\sigma - r_{ij}}{\rho_{ij}}\right)) - \frac{c_{ij}}{r_{ij}^6}$$
$$+ D_{ij}\{\exp[-2\alpha_{ij}(r_{ij} - r_{ij}^0)] - 2\exp[-\alpha_{ij}(r_{ij} - r_{ij}^0)]\}$$

	A_{ij} (eV)	ρ_{ij} (Å)	σ_{ij} (Å)	C_{ij} (eV.Å^{12})
Mg−O	0.00772258975333	0.178000	2.71550	10.91690808
Si−O	0.00707180971794	0.163000	2.36830	0
O−O	0.01197435265120	0.276000	3.54000	27.29493416

	D_{ij} (eV)	α_{ij} (Å^{-1})	r_0 (Å)
Mg−O	0.436800	2.0000	1.75
Si−O	0.655200	2.0000	1.47

Pedone2006 (Pedone et al., 2006) $q_{\mathrm{O}} = -1.2e$

$$U(r_{ij}) = \frac{q_i q_j}{r_{ij}} + D_{ij}\{[1 - \exp(a_{ij}(r_{ij} - r_0))]^2 - 1\} + \frac{C_{ij}}{r_{ij}^{12}}$$

	D_{ij} (eV)	a_{ij} (Å^{-2})	r_0 (Å)	C_{ij} (eV.Å^{12})
Mg−O	0.038908	2.281000	2.586153	5.0
Si−O	0.340554	2.006700	2.100000	1.0
O−O	0.042395	1.379316	3.618701	22.0

Dufils2017 (Dufils et al., 2017) $q_{\mathrm{O}} = -0.945e$

$$U(r_{ij}) = \frac{q_i q_j}{r_{ij}} - A_{ij} \exp\left(-\left(\frac{r_{ij} - l_{ij}}{\lambda}\right)^2\right) + B_{ij} \exp\left(\frac{-r_{ij}}{\rho_{ij}}\right) - \frac{C_{ij}}{r_{ij}^6}$$

	A_{ij} (eV)	l_{ij} (Å)	B_{ij} (eV)	ρ (Å)	C_{ij} (eV.Å^6)
Mg−O	33951.076	2.00	33951.076	0.18000	40.552288
Si−O	50466.337	1.61	50466.337	0.15700	31.471009
O−O	1585.734	0	0	0.32500	54.514310

Table 1: Potential energy functions and parameters for the semi-empirical potentials compared in the present study. The cation charges are $q_{\mathrm{Mg}} = -q_{\mathrm{O}}$ and $q_{\mathrm{Si}} = -2q_{\mathrm{O}}$.

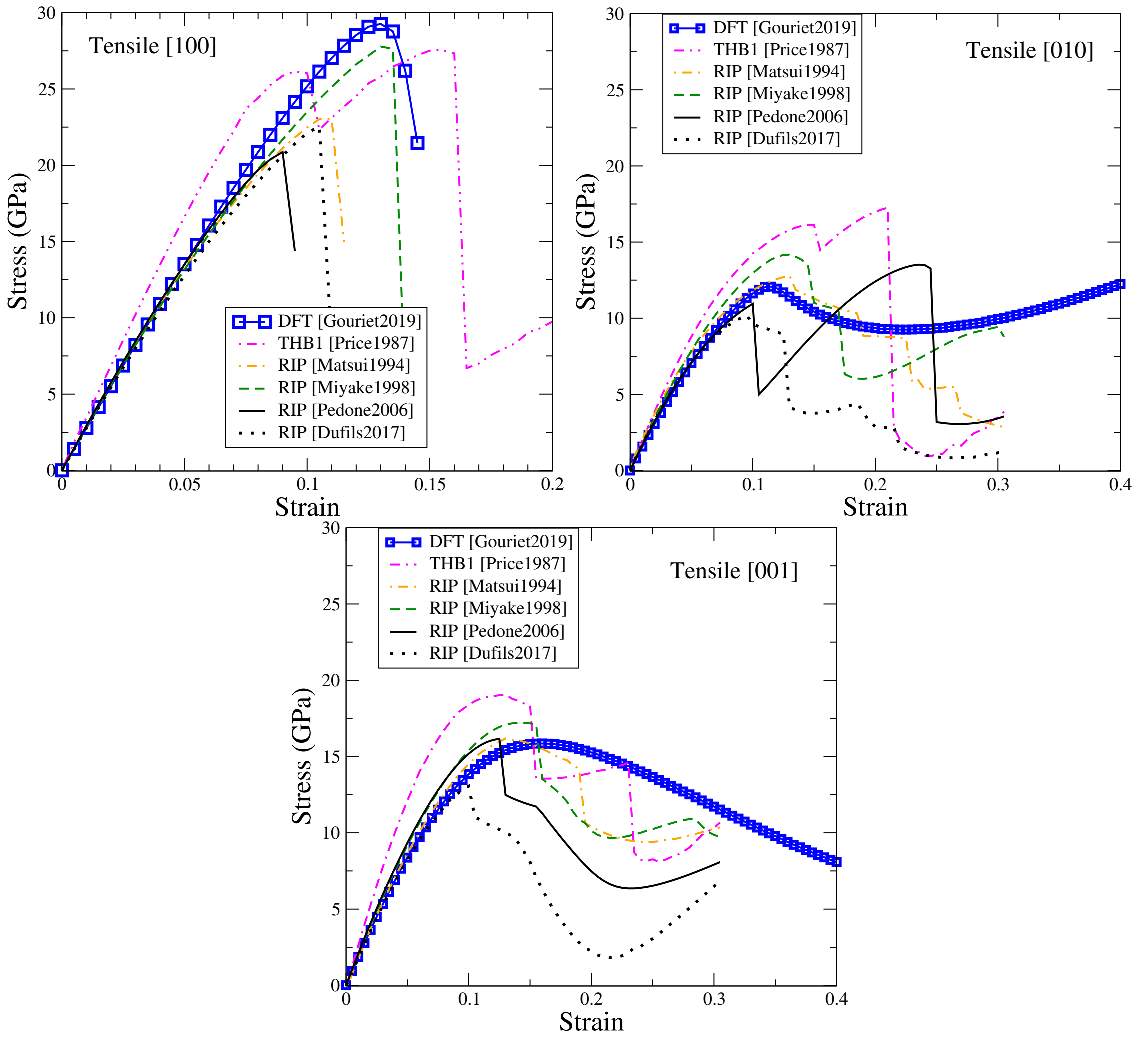

Figure 1: Stress-strain curves obtained in pure tension.

its derivative (i.e. forces acting on atoms) up to about 10% deformation, before deviating from DFT and reaching instability.

The stress-strain curves obtained during shear deformation are reported in Fig. 2. Again the fact that the potentials match the slope of the DFT curve at small deformations translate their ability to reproduce the stiffness constants. Depending on the solicitation axis, interatomic potentials match DFT up to 10 to 20% shear strain, which is quite remarkable considering the simplicity of these models. As for tension, instability is often marked by a sharp drop in stress.

3. (010) stacking fault

As presented in the main article, rigid-ion potentials fail to reproduce the generalized stacking fault energies in the (010) plane. The stacking fault (SF) corresponding to a translation of 1/2[001] in the (010)

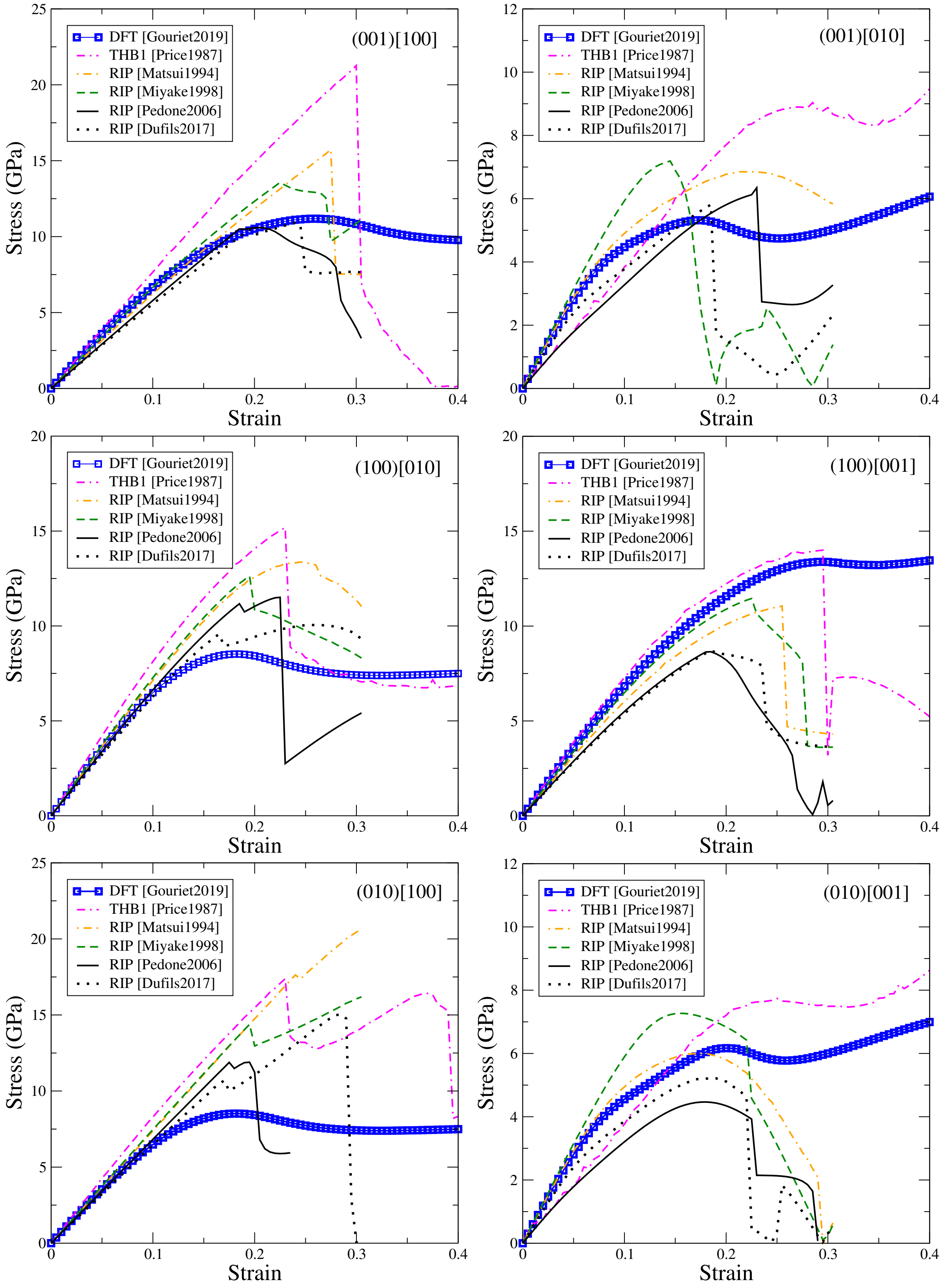

Figure 2: Stress-strain curves obtained during shear deformation.

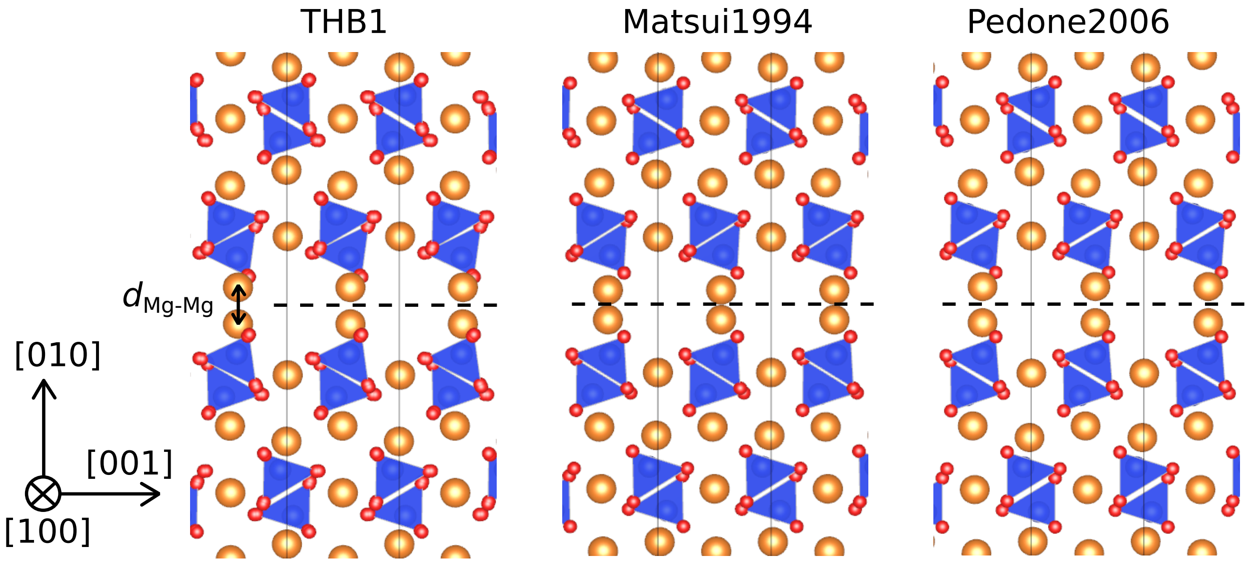

Figure 3: Atomic configurations of the 1/2[001](010) stacking fault obtained with three different potentials: THB1 (left), Matsui1994 (middle), Pedone2006 (right). The Miyake1998 potential produces a configuration similar to the Matsui1994 potential, while the Dufils2017 produces one similar to Pedone2006. The plane of the stacking fault is indicated by a dashed line. The distance $d_{\text{Mg-Mg}}$ is defined as the distance between Mg ions apart from the stacking fault. Note that the two Mg ions do not belong to the same plane: one ion is actually shifted by 1/2[100] along the direction normal to the figure.

plane should correspond to a maximum of the energy and be unstable, however most rigid-ion potentials produce a local energy minimum. We present here the atomic configurations corresponding to this SF.

The five interatomic potentials produce very similar lattice constants (Fig. 2), hence also similar distances between neighbouring Mg ions; this distance in bulk forsterite is about $d_{\text{Mg-Mg}} \approx 3.89$ Å. In the (010)[001] SF Mg ions become closer to one another, as shown in Fig. 3. After relaxing ions in the direction normal to the plane, the distance between Mg ions become $d_{\text{Mg-Mg}} \approx 3.39$ Å with the THB1 and Pedone2006 potentials; with the Matsui1994 and Miyake1998 potentials, it is reduced to $d_{\text{Mg-Mg}} \approx 3.22$ Å.

We note that all interatomic potentials produce similar SF configurations, therefore the problem is not due to a difference in the relaxation nor in the final state. The energy difference comes primarily in the potential function, and since Mg ions interact with one another only through the Coulomb interaction, it is the main source of error in the computation of the energy. Indeed, potentials where Mg ions have a large charge (THB1, Pedone) produce a large penalty energy when Mg ions are close to one another, while potentials with small partial charges (Matsui1994, Miyake1998) result in a smaller penalty. This is consistent with the fact that the latter erroneously produce a local energy minimum for this SF.

4. Parent oxide phases

As discussed in the manuscript, the ability of interatomic potentials to describe Schottky defects (and possibly other defects) in forsterite depends on their transferability to the parent oxide phases, namely

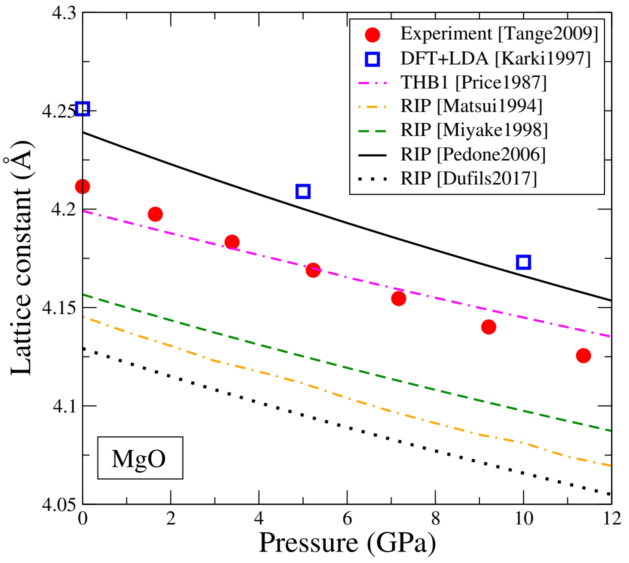

Figure 4: Lattice constant of MgO with the cubic rock-salt lattice as function of pressure.

magnesium oxide (MgO) and silicon dioxide (SiO_2). In this section we present our computations of the bulk lattice properties of these two phases.

4.1. Magnesium oxide

In the whole pressure range studied, magnesium oxide MgO crystallizes in the rock-salt lattice. The bulk properties of this ceramic are well characterized and published in many experimental and numerical studies.

The pressure dependence of the lattice constant of MgO is reported in Fig. 4. Values from the literature reveal that DFT+LDA calculations overestimate the lattice constant, as is common with this method. Two interatomic potentials produce lattice constants within the range of experimental and DFT data: the THB1 and Pedone2006 potentials, which is sensible given that both were parametrized using the properties of MgO. The other interatomic potentials underestimate the lattice constant by 1 to 2%, which is still acceptable.

MgO being cubic, its elastic behaviour is characterized by only three constants, namely c_{11}, c_{12} and c_{44}. Fig. 5 shows their evolution with pressure as computed with interatomic potentials. The THB1 potential overestimates c_{11} and c_{12}, but is in best agreement with experiment and DFT for c_{44}. The Matsui1994, Miyake1998 and Dufils2017 potentials overestimate c_{11} by 30% or more, and largely underestimate c_{44}. The Pedone2006 potential provides the best match for c_{11} and c_{12}, although it underestimates c_{44} by about 50%.

4.2. $\alpha-$quartz SiO_2

In the pressure range from 0 to 12 GPa, silicon dioxide SiO_2 crystallizes preferentially in the α-quartz phase, therefore we consider only this phase in the present study. Quartz is a crystal of hexagonal symmetry, where SiO_4 tetrahedra are connected by their tips.

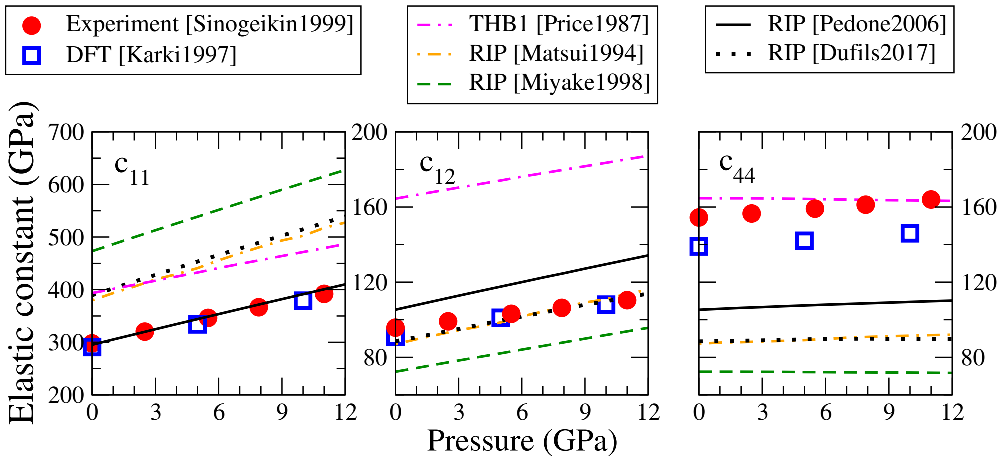

Figure 5: Elastic constants of MgO as function of pressure. Other elastic constants are such that $c_{11} = c_{22} = c_{33}$, $c_{12} = c_{13} = c_{23}$, and $c_{44} = c_{55} = c_{66}$.

Fig. 6 gives the two lattice constants a and c of quartz as function of pressure. As in the other phases, the DFT+LDA calculations (blue empty squares) underestimate the lattice constants with respect to experimental values (filled red discs). The various interatomic potentials perform differently. The THB1, Matsui1994 and Pedone potentials are the closest to experimental values. The Dufils2017 potential largely overestimates both lattice constants, probably because it was designed to model melts where Si−O bonds are longer and softer than in crystals.

The six independent elastic constants of α-quartz are reported in Fig. 7. Again, the THB1, Matsui1994 and Pedone2006 potentials are in close agreement with experimental and DFT+LDA values. The potentials Miyake1998 and Dufils2017 predict negative values of c_{12} at ambient pressure, i.e. they predict that quartz would not be stable at ambient pressure. They perform better at pressures greater than 4 GPa.

The behaviour of the c_{66} component is interesting: experimentally its value decreases up to 5 GPa, and then increases again. This behaviour is well reproduced by the Matsui1994 and Pedone2006 potentials, however the THB1 and Dufils2017 potentials predict a monotonous decrease over the whole pressure range studied.

References

Dufils, T., Folliet, N., Mantisi, B., Sator, N., Guillot, B., 2017. Properties of magmatic liquids by molecular dynamics simulation: The example of a MORB melt. Chem. Geol. 461, 34–46. doi:10.1016/j.chemgeo.2016.06.030.

Gouriet, K., Carrez, P., Cordier, P., 2019. Ultimate Mechanical Properties of Forsterite. Minerals 9, 787. URL: https://www.mdpi.com/2075-163X/9/12/787, doi:10.3390/min9120787.

Matsui, M., 1994. A Transferable Interatomic Potential Model for Crystals and Melts in the System CaO-MgO-Al2O3-SiO2. Mineral. Mag. 58A, 571–572. doi:10.1180/minmag.1994.58a.2.34.

MIYAKE, A., 1998. Interatomic potential parameters for molecular dynamics simulation of crystals in the system K2O-Na2O-

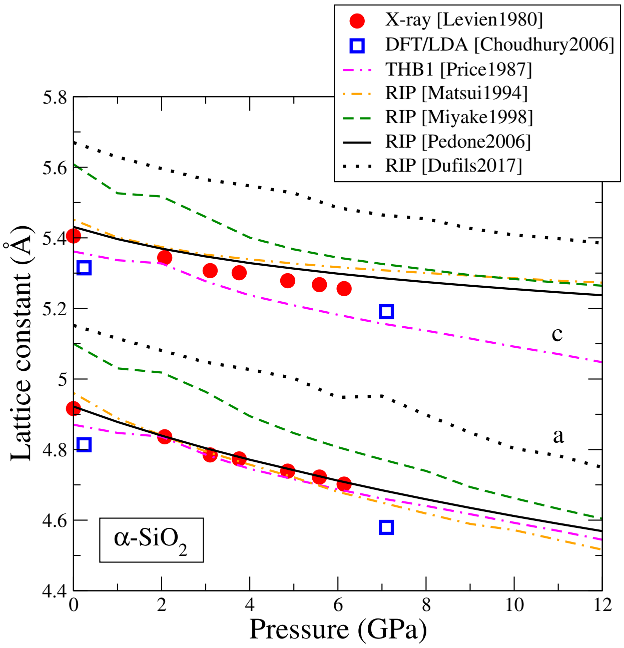

Figure 6: Lattice constant of α-quartz SiO_2 as function of pressure.

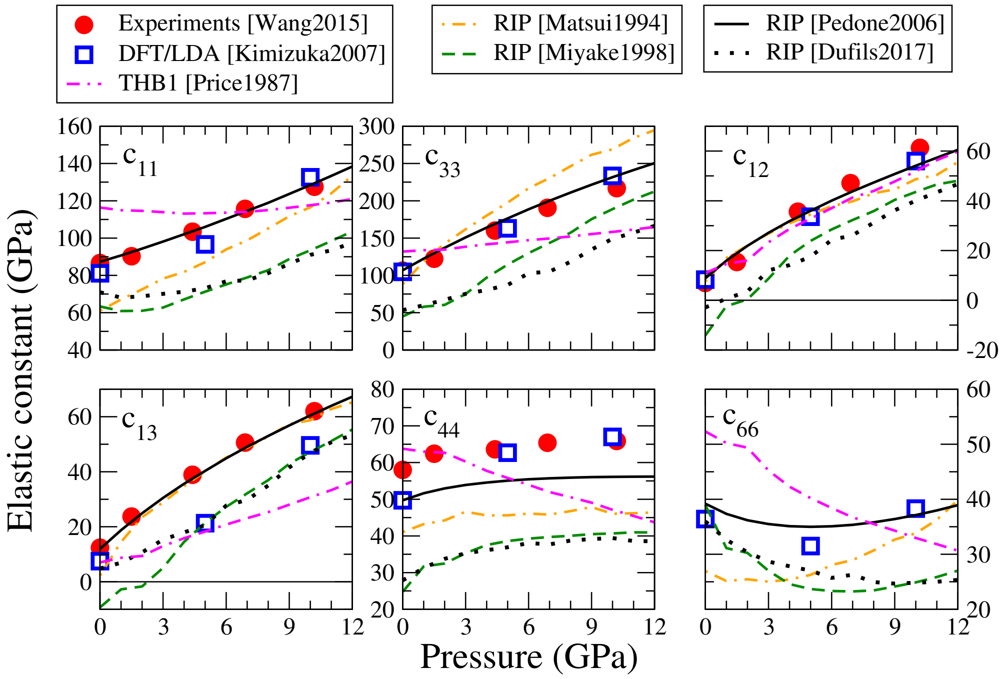

Figure 7: Elastic constants of α-quartz SiO_2 as function of pressure.

CaO-MgO-Al2O3-SiO2. Mineral. J. 20, 189–194. URL: https://www.jstage.jst.go.jp/article/minerj/20/4/20_4_189/_pdfhttp://www.jstage.jst.go.jp/article/minerj/20/4/20_4_189/_article, doi:10.2465/minerj.20.189.

Pedone, A., Malavasi, G., Menziani, M.C., Cormack, A.N., Segre, U., 2006. A New Self-Consistent Empirical Interatomic Potential Model for Oxides, Silicates, and Silica-Based Glasses. J. Phys. Chem. B 110, 11780–11795. URL: https://pubs.acs.org/doi/10.1021/jp0611018, doi:10.1021/jp0611018.

Price, G.D., Parker, S.C., Leslie, M., 1987. The lattice dynamics and thermodynamics of the Mg2SiO4 polymorphs. Phys. Chem. Miner. 15, 181–190. doi:10.1007/BF00308782.
